# The pharmacogenetics of the new-generation antipsychotics – A scoping review focused on patients with severe psychiatric disorders

**DOI:** 10.3389/fpsyt.2023.1124796

**Published:** 2023-02-16

**Authors:** Octavian Vasiliu

**Affiliations:** Department of Psychiatry, Dr. Carol Davila Central Military Emergency University Hospital, Bucharest, Romania

**Keywords:** brexpiprazole, aripiprazole, cariprazine, lumateperone, pimavanserin, pharmacokinetics, pharmacogenetics

## Abstract

Exploring the possible correlations between gene variations and the clinical effects of the new-generation antipsychotics is considered essential in the framework of personalized medicine. It is expected that pharmacogenetic data will be useful for increasing the treatment efficacy, tolerability, therapeutic adherence, functional recovery, and quality of life in patients with severe psychiatric disorders (SPD). This scoping review investigated the available evidence about the pharmacokinetics, pharmacodynamics, and pharmacogenetics of five new-generation antipsychotics, i.e., cariprazine, brexpiprazole, aripiprazole, lumateperone, and pimavanserin. Based on the analysis of 25 primary and secondary sources and the review of these agents’ summaries of product characteristics, aripiprazole benefits from the most relevant data about the impact of gene variability on its pharmacokinetics and pharmacodynamics, with significant consequences on this antipsychotic’s efficacy and tolerability. The determination of the CYP2D6 metabolizer status is important when administering aripiprazole, either as monotherapy or associated with other pharmacological agents. Allelic variability in genes encoding dopamine D2, D3, and serotonin, 5HT2A, 5HT2C receptors, COMT, BDNF, and dopamine transporter DAT1 was also associated with different adverse events or variations in the clinical efficacy of aripiprazole. Brexpiprazole also benefits from specific recommendations regarding the CYP2D6 metabolizer status and the risks of associating this antipsychotic with strong/moderate CYP2D6 or CYP3A4 inhibitors. US Food and Drug Administration (FDA) and European Medicines Agency (EMA) recommendations about cariprazine refer to possible pharmacokinetic interactions with strong CYP3A4 inhibitors or inducers. Pharmacogenetic data about cariprazine is sparse, and relevant information regarding gene-drug interactions for lumateperone and pimavanserin is yet lacking. In conclusion, more studies are needed to detect the influence of gene variations on the pharmacokinetics and pharmacodynamics of new-generation antipsychotics. This type of research could increase the ability of clinicians to predict favorable responses to specific antipsychotics and to improve the tolerability of the treatment regimen in patients with SPD.

## 1. Introduction

Exploring the correlation between genotype and clinical response to the administration of antipsychotics is an investigation motivated by many intricated factors, with both theoretical and pragmatic aims. The need to find pharmacogenetic prognosis variables for obtaining a higher efficacy seems the most obvious reason for this exploration, but this is far from being the only direction of research. Clinicians are also interested in detecting possible associations of allelic variations and haplotype with treatment tolerability, which correlates with therapeutic adherence, quality of life, involvement of the patient in functional recovery, etc (1, 2). *Pharmacogenetics* is defined as “the study of genetic causes of individual variations in drug response” and it mainly explores the relationship between individual genetic factors and the efficacy/tolerability of a specific pharmacologic agent (3). *Pharmacogenomics* is focused on investigating “the simultaneous impact of multiple mutations in the genome that may determine the patient’s response to drug therapy” (4). Otherwise formulated, pharmacogenomics represents an application of pharmacogenetics to the whole genome and it may be considered a step forward in the characterization of gene-drug interactions (5). This is important for creating a personalized medicine that should involve not only searching for single gene effects but integrating the impact of the whole genome on the pharmacokinetics, pharmacodynamics, safety, and tolerability of drugs (5).

The interest in exploring pharmacogenetics and pharmacogenomics of severe psychiatric disorders (SPDs) has been documented in an impressive number of papers covering more than two decades of research (6, 7). An early analysis of the genomewide significance for the effects of antipsychotics included 738 patients from the Clinical Antipsychotic Trials of Intervention Effectiveness (CATIE), and the results supported the existence of relevant single nucleotide polymorphisms (SNPs), e.g., ANKS1B and CNTNAP5 as mediators of olanzapine and risperidone effects on negative symptoms (6). The most significant associations in psycho-pharmacogenetics, at least until now, remain those between the drug metabolic polymorphisms of cytochrome P450 isoenzymes, especially CYP2D6 and 2C19, and the onset of treatment responsiveness or adverse events (7, 8).

Establishing a phenotype of schizophrenia (based on genetic, neuro-imagistic, biochemical, clinical, and other variables) for effective response or increased tolerability to certain antipsychotic(s) would presumably involve significant pharmacoeconomic benefits (9). Pharmacogenetic testing has been associated with “real-world” cost savings and improvement in adherence in patients who switched or added a new psychotropic medication after a failed monotherapy trial (9). Treatment tolerance and adherence are influenced by pharmacogenomics variables, e.g., the HTR2C gene and the risk of metabolic dysregulation, MC4R and OGFR1 genes for weight gain, CYP2D6 and the risk of extrapyramidal symptoms (EPS) or tardive dyskinesia, etc (10). The evidence on the cost-effectiveness of genome-guided therapeutic strategies in psychiatric disorders is growing, but the need to intensify the efforts on economic evaluations of pharmacogenomic implementation in psychiatry has been reported by different authors (11).

Another important aspect of pharmacogenetic research is the possibility of anticipating the consequences of concomitantly administering multiple drugs, which is far from being an isolated phenomenon in patients with SPDs. Polypharmacy was found in 85% of the hospitalized patients enrolled in a study (*N* = 320 participants diagnosed with schizophrenia spectrum disorders (SSD) or depressive disorders (12). On average, these patients received 4.5 ± 2.7 medications, out of which 3.3 ± 1.8 were psychotropics (12). Adverse events of the administered drugs were 2–3 times more frequent than in the case of monotherapy (12). Multiple combinations of antidepressants, antipsychotics, mood stabilizers, anxiolytics, hypnotics, and anticholinergics are concomitantly administered with different drugs for somatic or psychiatric comorbidity or as augmentative agents for the main diagnosis (12–14). Even more, the trend toward prescribing multiple concomitantly administered psychotropics was confirmed by pharmacoeconomic studies at a significant level: visits with ≥2 medications increased from 42 to 60% in a decade (1996–2006), while visits with ≥3 medications increased from 17 to 33% in the same period (15). Prescriptions for at least two antidepressants, antipsychotics, sedative-hypnotics, and antidepressant-antipsychotic combinations significantly increased in a 10-year timeframe (15). This phenomenon of polypharmacy could, at least at a theoretical level, be avoided if an evidence-based treatment is initiated from the very beginning, including pharmacogenetic variables besides other biological and clinical parameters. An overview of 42 pharmacological co-treatment strategies added to antipsychotic treatment in patients with schizophrenia and sub-optimal response -explored in 29 meta-analyses (*n* = 381 trials, *N* = 19833 participants) -concluded there was a low quality of the studies investigated and a low level of evidence level for all combined interventions, except for non-steroidal anti-inflammatory drugs (16). Although epidemiological studies demonstrated that polypharmacy is an extremely common occurrence in patients with schizophrenia, clinical trials supporting the efficacy of specific drug combinations are very few (17). Associations of different antipsychotics are not supported by evidence; antidepressant administration in this population does not benefit from consistent support from randomized clinical trials but may reduce the suicide rate and all-cause mortality, while benzodiazepines have associated risks of increased mortality in observational studies (18). Pharmacogenetics possesses the potential to optimize antipsychotic treatment by predicting clinical outcomes and reducing the “trial-and-error” strategies (19), although good quality trials to determine this hypothesis are yet needed.

Treatment-resistant schizophrenia is another pathology that could benefit from the discoveries in the field of pharmacogenetics (20, 21). The relationship between antipsychotic response and neurodevelopmental candidate genes in treatment-resistant schizophrenia is currently being explored, 21, 22). The genetic modeling of pharmacokinetics and pharmacodynamics of antipsychotic agents is a component of interest for personalized medicine (21, 22). In a retrospective analysis, the importance of therapeutic drug monitoring and pharmacogenetics of antipsychotics and antidepressants during 5 years was assessed in real-life settings (23). The results of this study suggest that physicians are becoming more confident in the utility of pharmacogenetic testing and drug-level monitoring, i.e., in the rational selection of the most appropriate drug and dose for a certain patient (23). Regarding the use of pharmacogenetic testing in patients receiving antipsychotics, it was concluded that clozapine, haloperidol, and aripiprazole were the most frequently associated with recommendations for this type of investigation (23). The efficacy of pharmacogenetic testing in clinical practice is supported by prospective studies showing, for example, that mean scores of mental quality of life, depression severity, and various clinical outcomes improved when antidepressant treatment was guided by pharmacogenetic testing, and earlier testing ameliorated outcomes sooner (24).

The current state of pharmacogenetic testing in clinical practice is, however, very difficult to define due to both objective (e.g., high costs, lack of reimbursement from health insurance companies, limited accessibility, etc.) and subjective (e.g., lack of familiarity with this type of testing on the part of clinicians and patients, mistrust about the utility of genetic testing in daily practice, etc.) factors. Although the number of commercial companies worldwide producing pharmacogenomic test kits is increasing, each kit is different in the number and variety of the genes tested and the variants they are intended to screen for (25). Therefore, the reports resulting from the administration of such pharmacogenetic tests may be quite heterogenous, based on the evidence included or excluded by the manufacturers, leading to discordant phenotype analysis when tests originated in distinct companies are comparatively analyzed (25). This indicates the need for continuous research dedicated to the validation of different gene-drug interactions and to the creation of consensus-based pharmacogenetic/pharmacogenomic evaluation strategies, in order to increase the homogeneity of the results and the possibility of finding the most appropriate monitoring tools for patients with SPD.

The perception of pharmacogenetic testing in clinicians needs to be constantly assessed and educating psychiatrists about the potential benefits of guiding their treatment on genetic factors may improve the quality of case management for patients with psychiatric disorders. In a survey, more than 80% of the psychiatrists acknowledged that pharmacogenomic testing would be beneficial for identifying the most appropriate treatments, while more than 70% considered these tests would be useful for predicting medication intolerance (26). Treatment-refractory cases, patients with low tolerability to previously administered drugs, and subjects who need drug monitoring throughout their therapy, all may benefit from pharmacogenomic testing. Although mental health specialists may be aware of pharmacogenomic testing, they may not be informed about all the advantages and limitations of this method. Therefore, increasing their awareness about pharmacogenomic testing can be very useful for patients with SPD, because, as stated above (25), implementing this type of testing can improve the quality of life, responsivity rate, and tolerability of psychiatric treatment.

## 2. Pharmacogenetic modeling of the clinical effect during antipsychotic treatment

The pharmacokinetics of antipsychotic drugs is influenced by genetic factors (27), and knowing the genotype of cytochrome P 450 isoenzymes (CYP450) could be beneficial for the detection of potential causes of treatment non-responsivity or toxicity. Different SNPs in the genes CYP1A2, CYP2D6, and 3A4/5 have been shown to influence the pharmacokinetics of atypical antipsychotics (e.g., olanzapine, clozapine, aripiprazole, risperidone, quetiapine) (27). Besides CYP 450 system, other important factors that could modulate the responsivity to administered drugs are the transport systems, e.g., P-glycoprotein (P-gp) or multidrug resistance protein 1 (MDR1), breast cancer resistance protein (BCRP), etc., which are active efflux transporters in the blood-brain-barrier (27, 28).

The relationship between the metabolizer status, determined by the CYP 450 genotype, and the clinical response to medication is not linear because of the phenomenon called “phenoconversion” (29). This represents the transformation of extensive genotypic metabolizers into phenotypic poor metabolizers due to extrinsic factors (29). Phenoconversion is responsible for the genotype-phenotype mismatch in extensive metabolizers and explains why the number of phenotypic poor metabolizers may be higher than the number predicted by genotyping (29). Pharmacological agents can inhibit CYP450 enzymes, thus leading to a decrease in functioning and poor metabolizer-like status, or they can induce these enzymes and mimic an extensive metabolizer status (29). The phenoconversion depends on the dose and duration of the co-administered drugs acting on the same isoenzyme (29).

The influence of genetic variables on the pharmacodynamics of antipsychotic drugs is another topic of major interest for researchers and clinicians. This modulatory effect has been mainly explored in relation to the risk of various adverse events, but research is trying to correlate genetic background with the probability of achieving a higher rate of response to antipsychotics (30). Therefore, mutations in genes for dopamine and serotonin receptors, secondary messengers, enzymes involved in the degradation of monoamines, neurohormones, and inflammatory markers are extensively investigated in relation to the clinical effects of antipsychotics (30).

Both pharmacokinetic and pharmacodynamic parameters are influenced by variations in a unique SNP or combinations of SNPs (called haplotype) localized in genes responsible for encoding essential proteic structures within and outside the central nervous system (CNS) (Table 1) (31). A higher number of genetic variants measured within a haplotype correlates with a more precise determination of the alleles involved (31), and this could, in turn, lead to a more precise investigation of the genotype-to-phenotype correlation. According to the currently available data, more than 170 CYP2D6 allelic variants (from CYP2D6*1 to CYP2D6*171), 39 CYP2C19 gene variants, 37 CYP3A4 genetic variations, and more than 20 variants of the CYP1A2 gene (from CYP1A2*1A to CYP1A2*21, plus a number of SNPs with undetermined haplotype) have been identified (32). These numbers only illustrate the genetic modulation of the most important isoenzymes at the CYP450 level, but there are SNPs influencing gene expression for almost every previously mentioned factor involved in the pharmacokinetics and pharmacodynamics of the antipsychotics (i.e., receptors, transporters, etc.) (32).

**TABLE 1:** The most explored pharmacogenetic targets for antipsychotics efficacy and tolerability (19, 34, 54, 56, 59, 60).

Gene (localization)	Encoded protein(s), most frequent alleles	Role in the PG of antipsychotics
CYP1A2 (15q24.1)	CYP1A2, *1A (wild allele), *1C, *1F, *1K	*1F- UM(↑), *1C and *1K- PM(↑) Clozapine and olanzapine are substrates of CYP1A2.
CYP2C19 (10q23.33)	CYP2C19, *1 (wild allele), *2, *17	*2- LOS allele, *17- EF allele Clozapine is a substrate for this isoenzyme.
CYP2D6 (22q13.2)	CYP2D6, *1 (wild type), *2, *3, *4, *5, *6, *10, *11, *22, *25	*1/*1 x N or *2/*2 x N genotype- UM; *1/*10, *1/*1, *1/*2 genotype- NM; *4/*10, *10/*11, *1/*5 genotype- IM; *3/*4, *4/*4, *5/*6 genotype- PM Aripiprazole, clozapine, haloperidol, quetiapine, risperidone, brexpiprazole, cariprazine, and pimavanserin are substrates.
CYP3A4 (7q21-q22.1)	CYP3A4, *1 (wild type), *1B, *1G, *4, *5, *22, *23 CYP3A5, *1 (wild type), *3, *6, *7	3A4*1B and 3A5*3- may influence the oral clearance of their substrates; 3A4*22- risperidone↓/↑%, aripiprazole and haloperidol- no impact, ↑% of quetiapine. Pimavanserin, lumateperone, aripiprazole, brexpiprazole, cariprazine, quetiapine, ziprasidone and other second-generation antipsychotics, but also many first-generation antipsychotics.
ABCB1 (7q21.12)	P-gp or MDRP1, SNP rs1045642 or C3435T	SNP rs1045642- ↓expression of MDRP1; ABCB1 3435TT- ↑% of clozapine; no impact on olanzapine and quetiapine; not significant for aripirpazole; significant relation with% of risperidone; 1236C and 3435 alleles- favorable response to antipsychotic treatment (haloperidol, clozapine, risperidone); 1236TT, 2677TT, and 3435TT genotypes- more frequent sedation or dizziness to antipsychotics (same as above)
SLC18A2 (10q25.3)	VMAT2	Could be a marker for tardive dyskinesia in patients treated with antipsychotics, but data is inconsistent.
GABRB1 (4p12)	GABA type A receptor subunit β1	↑antipsychotic dosage (replication studies are needed)
DRD2 (11q23.2)	Dopamine D2 receptor	Taq1A A1 carriers-↑% prolactin vs. non A1 carriers

EF, enhanced function; IM, intermediate metabolizer; LOS, loss of function; MDRP1, multi-drug resistance protein 1; NM, normal metabolizer status; PG, pharmacogenetics; P-gp, P-glycoprotein; PM, poor metabolizer status; UM, ultra-rapid metabolizer status; VMAT2, vesicle monoamine transporter type 2, %, concentration/plasma level.

The gene for CYP1A2 has a large interindividual phenotypic variability (from five to 15-fold) depending on sex, smoking or non-smoking status, concomitant medications, and genetic polymorphisms (33). This gene is located on chromosome 15q24.1 (34). CYP1A2*1A is considered the wild allele, while *1C and *1K alleles are associated with decreased *in vivo* enzymatic activity, and *1F correlates with increased enzymatic activity (33). Although the CYP1A2*1F genotype has been associated with an ultrarapid metabolizer status, this phenotype was detected only when an inducer was co-administered (e.g., smoking or heavy coffee use) (34). The decreased metabolism observed in individuals with CYP1A2*1C or *1K haplotype is still undergoing exploration in order to determine its clinical impact (34).

The CYP2C19 gene is located on chromosome 10q23.33, and multiple single nucleotide variants have been identified (35). The enzyme it codes is involved in the metabolism of psychotropic drugs, including antidepressants, benzodiazepines, and antiepileptics, but also in the biotransformation of endogenous compounds, e.g., neurotransmitters, steroid hormones, or fatty acids (36). Ultrarapid (i.e., two increased function alleles), rapid (i.e., one increased function and one normal function allele), normal (i.e., absence of increased or loss-of-function alleles), intermediate (i.e., one loss-of-function allele) and poor (i.e., two loss-of-function alleles) metabolizer phenotypes have been described (37). CYP2C19*1 is the fully functional, wild-type allele, CYP2C19*17 is an enhanced function gene, while CYP2C19*2 is the most frequently reported loss-of-function allele (38).

CYP2D6 isoenzyme is encoded by a highly polymorphic gene located on chromosome 22q13.2, and its alleles may be non-functional, lower functioning, normally functioning, or present increased function (39). The corresponding phenotypes are poor (no enzymatic activity), intermediate (decreased enzyme activity), normal, and ultrarapid (increased enzyme activity) metabolizers (39). The increased enzyme activity is usually caused by gene copy number variations, i.e., ≥1 functional gene copy (39). A genotype-to-phenotype translation method has been suggested by the Clinical Pharmacogenetics Implementation Consortium (CPIC) in order to facilitate the homogeneity of the results in pharmacogenomic studies, but it is not universally accepted or used by genetic testing laboratories (39). Each allele has been assigned an activity value from 0 to 1, or, more specifically, 0 for no function, 0.25 or 0.5 for decreased function, and 1 for normal function (40). CPIC considers an individual may possess a poor metabolizer phenotype corresponding to an activity score of 0 (e.g., genotypes *3/*4, *4/*4, or *5/*6), an intermediate metabolizer phenotype if the score is lower than 1.25 but above 0 (e.g., genotypes *4/*10, *10/*11, or *1/*5), a normal metabolizer phenotype for activity scores between 1.25 and 2.25 (e.g., genotypes *1/*10, *1/*1, or *1/*2), while ultrarapid metabolizers have an activity score > 2.25 (e.g., genotypes like *1/*1×N or *2/*2×N) (41). Also, a residual category of “indeterminate metabolizer” is reserved for individuals carrying one or two alleles with uncertain functions (e.g., genotypes *1/*22 or *1/*25) (40, 41). According to the Dutch Pharmacogenetics Working Group (DPWG), ultrarapid metabolizers have an activity score > 2.5, normal metabolizers between 1.5 and 2.5, intermediate metabolizers between 0.5 and 1, and poor metabolizers have a 0 activity score (41).

The genes encoding the CYP3A subfamily (4,5,7, and 43) are located on the 7q21-q22.1 chromosome (42). CYP3A gene polymorphisms have been associated with decreased function and, in the case of drugs that are metabolized exclusively by these enzymes, a higher normalized area under the curve (AUC) and a tendency to lower normalized clearance were reported if a mutated allele for CYP3A4 existed (42). Still, the impact of CYP3A4 polymorphisms on the pharmacokinetics of several explored antipsychotics (aripiprazole, olanzapine, and quetiapine included) was not significant, according to a study that enrolled 251 healthy volunteers (42). The high inducible potential of the CYP3A4 enzyme can explain this lack of impact on pharmacokinetics in heterozygous individuals (42). Fluoxetine and fluvoxamine are inhibitors of the CYP3A4 isoenzymes, and the inactivation of this system leads to complex and long-lasting pharmacokinetic interactions and toxicity (43). In the case of drugs with narrow therapeutic indices, the functionality of CYP3A isoenzymes is important, and the plasma levels of the respective pharmacological agents should be monitored if pharmacokinetic interactions are expected (43).

Another study (*N* = 57 healthy European and African-American subjects) explored the associations between CYP3A4 and CYP3A5 genotype and phenotype under physiological and induced situations (17). The most common polymorphisms, i.e., CYP3A4*1B, CYP3A5*3, CYP3A5*6, and CYP3A5*7 did not correlate with significant variability in the functionality of these enzymes (17). Oral clearance was the only parameter correlated with the presence of CYP3A4*1B and CYP3A5*3 polymorphisms (17).

According to a review that explored the impact of the CYP3A4*22 genotype in metabolizing different substrates, it appears that this relatively minor allele frequency (3–5% in the general population) has an impact on enzymatic activity (44). Regarding the antipsychotic metabolism, risperidone was associated with conflicting results when CYP3A4*22 polymorphism was analyzed, while aripiprazole and haloperidol were not significantly affected by the same genotype (17, 42–47). On the other hand, quetiapine had significantly higher plasma concentrations in the presence of the CYP3A4*22 genotype (45).

It is important to take into consideration the impact of multiple isoenzymes metabolizing the same drug when analyzing the association between a certain genotype and plasma concentrations. Antipsychotics metabolized by multiple CYP450 enzymes will be less sensitive to the genotypic variations of a certain isoenzyme (44).

ABCB1 gene (ATP-binding cassette subfamily B member 1, MDR1) is located on chromosome 7q21.12, and it encodes a transporter (an ATP-dependent efflux pump, P-gp or MDRP1) which is present in many tissues, including the blood-brain-barrier (48, 49). This location makes it extremely important for numerous substrates targeting the CNS, including atypical antipsychotics. A large number of SNPs were reported in the ABCB1 gene, the most explored being SNP rs1045642 or C3435T, which may influence the functionality of MDRP1 by decreasing the expression of this transporter (50, 51). This association is not unanimously accepted, and there are negative studies, also (52).

In a study (*N* = 75 patients), the carriers of the ABCB1 3435TT genotype presented 1.6-fold higher clozapine plasma levels vs. non-carriers, which was a significant impact on the pharmacokinetics of this antipsychotic (53). Another study (*N* = 473 healthy volunteers) found no association between this genotype and the pharmacokinetics of olanzapine and quetiapine, a significant interaction between T/T carriers and the pharmacokinetics of risperidone, while in the case of aripiprazole, the association exists but was not significant (50). Yet another study (*N* = 192 participants) suggested the existence of a strong association between 1236C and 3435 alleles and their homozygous genotype and the favorable response to the antipsychotic treatment (i.e., clozapine, haloperidol, risperidone) (54). Tolerability of the antipsychotic treatment was also correlated with ABCB1 genotypes, i.e., sedation and dizziness were more frequently reported in patients with 1236TT, 2677TT, and 3435TT genotypes (54).

Single nucleotide polymorphisms in the genes for COMT, DRD2/ANKK1, DRD3, HTR2A, and HTR2C are the most explored in relation to the pharmacodynamics of antipsychotic medication (55). Also, polymorphisms in the GABRB1 gene (encoding the GABA type A receptor subunit β1) have been significantly correlated with higher antipsychotic dosage in an analysis that included 109 SNPs from 29 candidate genes (56). However, the use of polygenic risk scores (PRS) for predicting the antipsychotic dosage did not prove useful, which indicates that the configuration of a clinically relevant phenotype based on risk genes is still an insufficiently validated strategy (57, 58).

The tolerability profile was also explored in relation to various genotypes, e.g., the body weight increase during antipsychotic treatment was correlated with SNPs from ADRA2A (adrenoreceptor alpha-2A), ADRB3 (adrenoreceptor beta 3), BDNF (brain-derived neurotrophic factor), DRD2, GNB3 (guanine nucleotide-binding protein), HTR2C, INSIG2 (insulin-induced gene 2), MC4R (melanocortin-4 receptor), and SNAP25 (synaptosomal-associated protein, 25kDa) (61). Hyperprolactinemia and DRD2 Taq1A genotype may be correlated in patients with schizophrenia, according to a meta-analysis that included 15 studies; this report could not find the correlation to be consistent in all patients, although Taq1A A1 carriers had significantly higher prolactin plasma level than A1 non-carriers (59). Also, SLC18A2 could be a marker for the risk of tardive dyskinesia (TD) in patients undergoing antipsychotic treatment, but the data are yet inconsistent (19, 60). Other potential risk factors for TD have been localized in the HSPG2, DPP6, MTNR1A, SLC18A2, PIP5K2A, and CNR1 genes (60).

## 3. Objectives

This review explored the evidence that supports the influence of genetic variations in the modulation of clinical effects of the new-generation antipsychotics in patients with SPD, with the purpose of describing the current level of knowledge about this interaction.

## 4. Methods

For the purpose of this scoping review, the category of “new generation antipsychotics” included the D2/D3 receptors partial agonists (cariprazine, brexpiprazole, aripiprazole), but also lumateperone (which possesses D2 presynaptic partial agonism and postsynaptic antagonism), and pimavanserin (an antipsychotic agent with a very specific pharmacodynamic profile) (62–65).

Five electronic databases were searched (PubMed, Cochrane, Clarivate/Web of Knowledge, Google Scholar, and Semantic Scholar) for papers using the paradigm “third-generation antipsychotics” OR “new-generation antipsychotics” OR individual international non-proprietary names (INNs) of these agents AND “genetic variants” OR “pharmacogenetic” OR “pharmacogenomic” OR “allele variation” OR “pharmacokinetics” OR “pharmacodynamic.” All types of reports were included, both primary and secondary, and all patients with SPD, regardless of their age, were accepted for this review. The list of references for each reviewed paper was analyzed and supplementary searches were performed, if they were found within the aim of this paper. No inferior time limit was defined for the search, while the superior limit was established was December 2022.

Only reports on new-generation antipsychotics that evaluated genetic parameters in relation to clinical effects (efficacy, adverse events, toxicity) or pharmacologic parameters (pharmacokinetic variables- e.g., distribution, elimination, half-time, or pharmacodynamic- e.g., receptors occupancy, transporters efficacy) were allowed. Also, all summaries of product characteristics (SPCs) for the agents of interest were reviewed in order to find information about the pharmacologic and pharmacogenetics variables. Sources not containing clear reference to one of the new-generation antipsychotics were excluded, as were the papers without data regarding the pharmacokinetic, pharmacodynamic, or pharmacogenetic properties of the previously mentioned antipsychotics.

In the category of SPD were included mainly SSD, major depressive disorder (MDD), and bipolar disorders (BDs), without limitations regarding the phase of the disorder, the treatment administered, comorbid diseases, and the hospitalized or outpatient regimen. Studies and reviews including patients treated with new-generation antipsychotics but without a clear delimitation of the diagnoses or with other diagnoses than those previously presented were allowed, if reported on relevant data about pharmacologic or pharmacogenetics data. Also, studies reporting on healthy volunteers in a structured manner were allowed, as early-phase research could have significance if integrated into the larger context of the current research (e.g., control groups, descriptors of populational characteristics, etc.).

## 5. Results

Data about the pharmacokinetics, pharmacodynamics and pharmacogenetics characteristics of the new-generation antipsychotics was derived from the following sources: five reports on cariprazine (three reviews and two preclinical studies), three reports on brexpiprazole (two reviews and one preclinical study), 15 reports on aripiprazole (five reviews, nine clinical prospective or retrospective studies, one case report), one review on lumateperone, and one review on pimavanserin (Table 2). Besides the previously mentioned sources, ten SPCs were reviewed (six US Food and Drug Administration, FDA-approved and four European Medicines Agency, EMA-authorized), and three pharmacogenomic guidelines were also included in the analysis. Most of the clinical data were derived from patients with SSD (*N* = 219), alcohol use disorders (*N* = 94), bipolar disorders or SSD (*N* = 4), undisclosed diagnosis (*N* = 1334), and healthy volunteers (*N* = 196). All patients with undisclosed diagnoses were retrieved from only one source (66), focused on the evaluation of pharmacokinetic parameters in relation to the CYP2D6 genotype.

**TABLE 2:** Pharmacologic and pharmacogenetic characteristics of the new-generation antipsychotics.

Antipsychotic	Pharmacokinetics	Pharmacodynamic	Pharmacogenetics	Observations
Aripiprazole (67–71, 73–76, 79–87)	Metabolized by CYP3A4 and CYP2D6. P-gp is involved in the transportation of both the parent drug and its active metabolite. ↑% of CYP2D6 phenoconversion was reported in retrospective studies when concomitant inhibitors were administered. PM, but also IM and NM, were affected by this phenomenon. Phenoconversion was associated with ↑% of discontinuation in CYP2D6 PM due to adverse events. BMI changes were associated with CYP2D6 phenotype, the period of ARI administration, and the number of concomitant CYP2D6 substrates.	D2 receptor PA, high affinity for D3, 5HT1A, 5HT2A, 5HT2B, and 5HT7, moderate affinity for 5HT1D and 5HT2C receptors, low affinity for 5HT1B, 5HT3, and 5HT6 receptors; moderate affinity for α1A, α1B, α2A, α2C, and H1 receptors	CYP2D6 EM: half-time 75h vs. 146h in the case of CYP2D6 PM. CYP2D6 PM: ARI daily dose ↓ by 50% vs. NM (as recommended by the FDA). CYP2D6 PM was associated with a higher plasma level of parent drug + active metabolite than in CYP2D6 IM and also in IM vs. EM (at a trend level). CYP2D6 PM + strong CYP3A4 inhibitor: ARI daily dose ↓ by 75%. CYP3A5 and ABCB1 genotypes have been associated with differences in the PK of ARI. CYP3A5*1/*1 was associated with ↑% of dizziness. CYP2D6 IM or PM was associated with ↑% EPS in children. Nausea and vomiting were also associated with the CYP2D6 genotype *via* ↑ of the AUC. -141C Del variant of the D2 receptor: transition from ARI monohydrate to lauroxil might be indicated (case report). DRD2 Taq1 A1A1: more favorable response to ARI, but ↑ the risk for EPS. HTR2A-1438G/T102C GG/CC: may predict a lower rate of response to ARI for negative symptoms. HTR2A rs6313C + ADRA2A rs1800544 C/C vs. T/T or C/G: DBP-lowering effect in HV. DRD3 rs6280 Ser/Ser homozygotes: ↑% of akathisia. DRD2 rs1799732 G/-: ↑% of asthenia. CYP1A NM/RM: ↑% of insomnia. HTR2A rs6314 C/C and OPRM1 rs1799971 A/A: ↑% of somnolence. HTR2A rs6314 C/C: ↑ cholesterol level during ARI administration vs. T carriers. HTR2C rs3813929: ↑% of somnolence. DAT1/SLC6A3 9-repeat allele + functional polymorphisms COMT, DRD2, and DRD4 genes: ARI reduced ventral striatal activation and bar-lab drinking in AUD. CYP3A PM: ↑ PRL level vs. IM and EM. ABCB1 rs10280101 A/A, rs12720067 C/C, and rs11983225 T/T polymorphisms: ↑ PRL level vs. C, T, and respectively C allele carriers. COMT polymorphisms rs4680 G/G and rs13306278 T: influenced the% of the C-peptide after ARI treatment. BDNF rs6265 C/C: ↑ insulin levels during ARI treatment.	There are significant correlations between gene variability and PG and PD parameters, supporting the importance of PG evaluation in patients undergoing ARI treatment. Unfortunately, not all reviewed data showed relevant associations, and no phenotype for ARI favorable response/low risk of adverse events could be yet defined.
Brexpiprazole (3, 88–91)	Metabolized by CYP2D6 and CYP3A4, one active metabolite. CYP2D6 strong inhibitors: the antipsychotic dose ↓ by 50%. CYP3A4 strong/moderate inhibitors: the antipsychotic dose should ↓ by 75%. CYP3A4 strong/moderate inhibitor + CYP2D6 strong/moderate inhibitor: the antipsychotic dose should ↓ by 75%. Strong CYP3A4 inducers: titration of up to 2× daily maintenance dose of brexpiprazole in 1–2 weeks (not > 12 mg/day). No significant impact on OATP, OAT, OCT, MATE1, BSEP, P-gp, or BCRP.	D2 receptor PA, 5HT1A PA, antagonist of D3, 5HT2A, 5HT2B, 5HT7, α1A, α1B, α1D, H1, and M1 receptors	CYP2D6 PM: the antipsychotic dose needs to be ↓ by 50%; the exposure to brexpiprazole is 47% higher than in CYP2D6 EM. CYP2D6 PM + CYP3A4 inhibitors: 75% ↓ of the brexpiprazole dose is recommended. CYP2D6 EM + CYP3A4 inhibitor + CYP2D6 inhibitor: plasma concentration of brexpiprazole may ↑ 4–5× normal concentration; a 75% ↓ of the brexpiprazole dose is recommended.	Only data about the genetic modeling of the PK are available for brexpiprazole. More studies are needed to explore the impact of gene variability on the PD of this antipsychotic.
Cariprazine (98–101)	Metabolized by CYP3A4 and CYP2D6, two active metabolites. CYP3A4 potent inhibitors: ↓ by 50% total dose of cariprazine is recommended; if the inhibitors are discontinued, the dose of cariprazine needs to ↑. CYP3A4 inducers: not recommended in association with cariprazine. No significant interaction with P-gp, OATP1B1, OATP1B3, or BCRP.	D3-preferring D3/D2 receptor PA, 5HT1A receptor PA, antagonist of α1B, H1, and 5HT2C receptors	CYP2D6 metabolizer status was not associated with changes in the exposure levels for the parent drug or its active metabolites.	The data about the PG of cariprazine is derived from very few sources. More studies are needed in order to validate the current knowledge about the PG of this antipsychotic.
Lumateperone (63, 103)	Metabolized by CYP3A4, 2C8, and 1A2. This antipsychotic does not significantly interact with P-gp or BCRP. CYP3A4 inducers: if already initiated, then avoid starting lumateperone. If lumateperone is already initiated and strong CYP3A4 inhibitors are needed, then lumateperone is best to be replaced with another pharmacological agent. UGT inhibitors: avoid concomitant administration of lumateperone.	D2 presynaptic PA and postsynaptic antagonism, 5HT2A receptor antagonist, inhibitor of serotonin reuptake, and GluN2B-NMDA modulator	No relevant data about the PG of lumateprone has been found during the literature search.	One of the newest antipsychotics launched on the market, lumateperone, still needs extensive exploration of its PG.
Pimavanserin (64, 104)	Metabolized by CYP3A4, CYP3A5, CYP2J2, 2D6, and other CYP450 and FMO enzymes, a major metabolite exists. Transporters do not significantly intervene in the distribution of this antipsychotic. Strong CYP3A4 inhibitors: the dose of pimavanserin should ↓ by 50%. Possible reduced efficacy when this antipsychotic is coadministered with strong CYP3A4 inhibitors.	Inverse agonist/antagonist of 5HT2A and 5HT2C receptors, no significant affinity for dopaminergic receptors	No relevant data about the PG of pimavanserin has been found during the literature search.	The PG of this pharmacological agent was not yet systematically explored.

ARI, aripiprazole; AUC, area under the curve; AUD, alcohol use disorder; BCRP, breast cancer resistance protein; BMI, body mass inndex; BSEP, bile salt export pump; COMT, catechol-O-methyl-transferase; DBP, diastolic blood pressure; EM, extensive metabolizer status; EPS, extrapyramidal adverse effects; FMO, flavin-containing monooxygenase; HV, healthy volunteers; IM, intermediate metabolizer status; NM, normal metabolizer status; PA, partial agonist; PD, pharmacodynamics; P-gp, P-glycoprotein; PK, pharmacokinetics; PM, poor metabolizer status; RM, rapid metabolizer status; OATP1B1/B3, organic anion transporting polypeptides 1B1/1B3; BCRP, breast cancer resistance protein; OATP, organic anion transporting polypeptides; OAT, organic anionic transporter; OCT, organic cationic transporter; MATE1, multidrug and toxin extruder; PG, pharmacogenetics; PRL, prolactin; UGT, UDP-glucuronosyltransferase.

Aripiprazole has a high affinity for D2, D3, 5HT1A, 5HT2A, 5HT2B, and 5HT7 receptors, moderate affinity for 5HT1D and 5HT2C receptors, low affinity for 5HT1B, 5HT3, and 5HT6 receptors (67). Also, a moderate affinity of aripiprazole for adrenergic α1A, α1B, α2A, α2C, and histaminergic H1 receptors has been reported (67). This agent is included in the category of “D2/D3 partial agonists” because of its main action on D2 receptors, but it was also described as a full or moderate D2 receptor antagonist, in different preclinical paradigms (67). This is considered a specific feature of aripiprazole in translational studies because other antipsychotics have pharmacodynamic properties that remain stable in different testable biological conditions (67).

The liver extensively metabolizes aripiprazole through dehydrogenation, hydroxylation, and N-dealkylation (68). CYP3A4 and CYP2D6 are responsible for the dehydrogenation and hydroxylation of aripiprazole, while N-dealkylation is realized by the CYP3A4 (68). The active metabolite is dehydro-aripiprazole, and at a steady state, this metabolite represents 40% of the plasma drug concentration (68, 69). Extensive CYP2D6 metabolizers have a half-time for the elimination of aripiprazole of approximately 75 h, while in poor metabolizers, this parameter reaches 146 h (68). Aripiprazole and its active metabolite are substrates for P-gp (70). CYP3A5 and ABCB1 genotypes also have been correlated with differences in the pharmacokinetics of aripiprazole, with potential influence on the ratio of the adverse event (71).

In a retrospective study (*N* = 1334 aripiprazole-treated patients), clinicians reduced daily doses of the antipsychotic administered to CYP2D6 poor metabolizers by 15% (66). The incidence of switching from aripiprazole to other antipsychotics was not related to CYP2D6 metabolizer status in this study (66).

According to a meta-analysis (*n* = 10 studies, *N* = 649 participants), the plasma concentrations of aripiprazole were significantly different when extensive CYP2D6 metabolizers and intermediate metabolizers were compared, but not in the case of the intermediate vs. poor metabolizers (72). The levels of parent drug + active metabolite did not differ significantly between extensive, poor, and intermediate metabolizers, but at a trend level, plasma levels in poor metabolizers were higher than in the intermediate metabolizers, and in intermediate metabolizers vs. the extensive metabolizers (72).

Based on the FDA-approved oral and acute injectable aripiprazole label, concomitant administration of either strong CYP3A4 or CYP2D6 inhibitors with aripiprazole should indicate the need to decrease the dose of the antipsychotic by 50%, except when used as adjunctive treatment with antidepressants (68). If both strong CYP3A4 and CYP2D6 inhibitors are concomitantly administered, the dose of aripiprazole should be decreased to 25% of the intended dosage (68). When concomitant strong inducers of CYP3A4 are administered with aripiprazole, the antipsychotic dose should be doubled over 1 or 2 weeks (68). In poor CYP2D6 metabolizers, the recommended dose of aripiprazole by the FDA is 50% (68). Poor metabolizers who also receive strong CYP3A4 inhibitors should receive 25% of the intended dose (68). These recommendations are applicable to oral solution, tablets, orally disintegrating tablets, and acute injectable aripiprazole formulations (68).

The EMA-approved product label for oral aripiprazole also mentions the need to decrease the dose of this antipsychotic when coadministered with strong CYP3A4 or CYP2D6 inhibitors, and recommends the increase of aripiprazole dose when these inhibitors are withdrawn from the combined therapy (73). If CYP3A4 inducers are coadministered with aripiprazole, EMA also recommends the increase of the antipsychotic’s dose; if the inducer is withdrawn, then the aripiprazole dose should be reduced to its recommended dose (73). According to the European product label, in CYP2D6 poor metabolizers, concomitant use of strong CYP3A4 inhibitors may increase plasma concentrations of the antipsychotic compared to that in CYP2D6 extensive metabolizers, but no specific recommendation about the dose reduction is made (73).

According to the European product label for aripiprazole long-acting injectable (LAI) formulation, for known CYP2D6 poor metabolizers, if the clinician intends to initiate this treatment with a two-injection start, the administration should be done in two separate deltoid muscles or one deltoid and one gluteal muscle; the injection into two gluteal muscles is contraindicated (74). For poor CYP2D6 metabolizers, the starting dose should be 300 mg aripiprazole LAI for the one-injection start and continuation of oral aripiprazole for 14 days; if the two-injection start is preferred, the initial dose should be administered in two separate injections of 300 mg aripiprazole LAI along with one single dose of the previously administered dose of oral aripiprazole (74). If the patient is a CYP2D6-poor metabolizer and uses concomitantly a strong CYP3A4 inhibitor, the starting dose should be reduced to 200 mg for the one-injection start, and the two-injection start should not be used (74).

The maintenance dose of aripiprazole LAI should be adjusted if strong CYP2D6 or CYP3A4 inhibitors are used concomitantly for more than 14 days, i.e., a decrease to 300 or 200 mg monthly, if the intended dose was 400 or 300 mg monthly, respectively (74). If both strong CYP2D6 and CYP3A4 inhibitors are administered for more than 14 days, then the recommended doses of aripiprazole LAI are 200 or 160 mg monthly, for the intended doses of 400 or 300 mg monthly, respectively (74). The administration of CYP3A4 inducers for more than 14 days concomitantly with aripiprazole LAI is contraindicated (74). In case of adverse reactions despite dose adjustments of aripiprazole LAI, the necessity of concomitant use of CYP2D6 and CYP3A4 should be reassessed (74).

The same dose of 200 mg of aripiprazole monohydrate LAI is recommended for patients with CYP2D6 poor metabolizer status taking concomitantly CYP3A4 inhibitors by the FDA-approved product label (75). Also, in patients taking strong CYP2D6 or/and CYP3A4 inhibitors and in those receiving CYP3A4 inducers for more than 14 days, the FDA supports the same dosing recommendations and the same contraindications as the EMA (75).

In a retrospective study (*N* = 635 patients who received LAI or oral aripiprazole), both poor and intermediate metabolizers had significantly increased exposure to the aripiprazole and its active metabolite in the case of LAI and oral treatment (between 1.5 and 1.7 fold vs. normal metabolizers for LAI, and 1.6–1.7 for oral formulation) (76). Age did not significantly influence exposure to the parent drug and its metabolite (76).

In a case-control study (children 6–15 years old), individuals who received aripiprazole and developed EPS had a dysfunctional CYP2D6 phenotype, i.e., intermediate and poor metabolizers (77). An analysis that included 148 healthy volunteers from six bioequivalence studies who received a single dose of aripiprazole showed a direct correlation between the AUC of the antipsychotic and the rate of adverse events, especially nausea or vomiting, more frequently reported in women (71). The AUC of aripiprazole was also directly influenced by the CYP2D6 genotype, with a 50% increase in poor metabolizers vs. extensive metabolizers; also, the AUC of dehydro-aripiprazole decreased by 33% in the same conditions (71). Women and CYP3A5*1/*1 individuals showed more frequent dizziness, which could also be related to the ratio of active metabolite/parent drug changes dependent on the CYP3A5 genotype (71).

In a case report, oral, followed by the monohydrate LAI formula of aripiprazole, was initiated after failed trials of multiple antipsychotics in a 51-year-old patient with schizophrenia (78). A high dose of aripiprazole monohydrate LAI (400 mg every 3 weeks) still allowed exacerbation of psychosis 3 days before the next injection of the antipsychotic, which raised the question of resistance factors, including pharmacogenetic variables (78). The pharmacokinetic parameters possibly influenced by genetic factors have been excluded (78). Still, a -141C Del variant of the D2 receptor gene was detected, which has been previously associated with treatment resistance in schizophrenia (78). The transition to aripiprazole lauroxil LAI was initiated (882 mg every 4 weeks) and significantly improved the psychotic symptoms without exacerbations before the time of injection administration (78).

In a prospective trial, patients with SSD (*N* = 90) treated with aripiprazole had a differentiated response to the antipsychotic, based on their DRD2 Taq1 genotype (79). Patients presenting the A1A1 genotype had a more favorable response to aripiprazole, according to the evolution of the PANSS scores, and it is presumed that this may be the result of a higher occupancy of the D2 receptors in individuals with this genotype (79). Also, the changes in the EPS (assessed by Simpson-Angus Scale- SAS scores) were dependent on the genotype (A1A1 vs. A1A2 + A2A2), indicating a higher vulnerability for the A1A1 genotype for EPS (79).

The GG/CC genotype of the HTR2A-1438G/T102C polymorphisms may predict a lower response to aripiprazole for negative symptoms, according to a prospective study (*N* = 128 patients), that used PANSS scores as the primary outcome (80). This effect is influenced by the dosage of the antipsychotic, age, duration of the disease, and diagnostic subtype (80).

Aripiprazole was associated with a higher diastolic blood pressure lowering effect in healthy volunteers participating in a phase I trial who presented a polymorphism in the serotonergic 5HT2A receptors (HTR2A rs6313C genotype) and one in the α2 adrenergic receptors gene (ADRA2A rs1800544 C/C vs. T/T or C/G) (81). This observation may be explained by the participation of alpha-2 receptors and 5HT2A receptors in vasodilatation and sympathetic cardiovascular regulation (81).

The incidence of akathisia was higher in DRD3 rs6280 Ser/Ser homozygotes vs. Gly allele carriers and asthenia appeared more often in DRD2 rs1799732 G/- subjects vs. G/G homozygotes (81).

Another association was found between CYP1A2 NM/RM (normal metabolizers/rapid metabolizers) phenotype vs. UM (ultrarapid metabolizers) and reports of insomnia during aripiprazole administration (81). This observation is interesting because aripiprazole is not metabolized by CYP1A2 (81).

A retrospective chart review (*N* = 277 patients under 18 years old, mean age 14.3, who received oral aripiprazole) showed a high rate of phenoconversion (72.2%) due to concomitant administration of a CYP2D6 inhibitor (82). Most of this rate was explained by phenoconverted poor metabolizers, followed by normal metabolizers, intermediate metabolizers, and a very small percentage of ultrarapid metabolizers (48, 27, 24, and 1%, respectively) (82). Phenoconverted poor metabolizers discontinued treatment with aripiprazole due to adverse events (67%) more than any other groups (82). Body mass index (BMI) change was associated with CYP2D6 phenotype, the period of aripiprazole administration, and the number of concomitant CYP2D6 substrates (82).

Somnolence was detected more frequently in HTR2A rs6314 C/C (wild type) and OPRM1 rs1799971 A/A subjects vs. C/T or G carriers (81). Headache was reported more frequently in mutant allele carriers of HTR2C rs3813929 (81).

A placebo-controlled study evaluating the role of genetic variations in the dopaminergic system variations as moderators between aripiprazole administration and reward-related effects of alcohol patients with substance use disorder (*N* = 94 participants) included fMRI alcohol cue-related task and a bar lab paradigm (83). The dopamine transporter genotype evaluated was a variable tandem repeat polymorphism of the DAT1/SLC6A3 gene. This genotype moderated the antipsychotic effects vs. placebo, reducing the ventral striatal activation and bar-lab drinking only in carriers of the DAT1 9-repeat allele vs. 10R homozygotes (83). This genotype was previously correlated with lower expression of the dopamine transporter and higher reward-associated brain activation (83). In the same study, a composite score including the polymorphism of the DAT1/SLC6A3 gene and functional polymorphisms of catechol-O-methyl-transferase (COMT), DRD2, and DRD4 genes, supported a moderator effect on the medication: aripiprazole reduced the primary outcomes more in patients with alcohol use disorder who carried a larger number of genes associated with higher synaptic dopamine tone (83).

CYP2D6 and 3A phenotypes, as well as DRD3 and ABCB1 polymorphisms, are involved in modulating the prolactin plasma level variability in patients receiving aripiprazole (84). DRD2, HTR2A, and HTR2C genetic variants may influence the prolactin release in the case of second-generation antipsychotics but not during aripiprazole administration (84). Still, about 5% of the patients undergoing aripiprazole treatment may present hyperprolactinemia, and this may be related to the presence of a combination of rare polymorphisms not yet clearly identified (84).

The binding affinity of several atypical antipsychotics, aripiprazole included, for HTR2A depends on naturally occurring variations (85). For example, in an *in vitro* study, three polymorphic serotonin 2A receptors (Ile197Val, Ala447 Val, and His452 Tyr) were associated with significant changes in atypical antipsychotic affinity (86). Also, three polymorphisms (Tr25Asn, Ile197Val, and His452 Tyr) modified the antipsychotic potency (86).

CYP3A-poor metabolizers presented higher prolactin levels during aripiprazole treatment vs. intermediate and extensive metabolizers (87). ABCB1 rs10280101 A/A, rs12720067 C/C, and rs11983225 T/T polymorphisms were associated with significantly higher prolactin plasma levels vs. C, T, and, respectively C allele carriers (87). C-peptide concentrations were significantly higher after aripiprazole treatment compared to its initial levels, and COMT polymorphisms rs4680 G/G and rs13306278 T influenced these concentrations vs. A and C/C homozygotes, respectively (87). Regarding insulin levels, carriers of BDNF rs6265 C/C had greater increases in insulin levels compared to other genotypes under aripiprazole treatment (87). HTR2A rs6314 C/C subjects presented higher cholesterol concentrations when receiving aripiprazole vs. T allele carriers (87).

Brexpiprazole is a D2 receptor partial agonist, but with less intrinsic activity than aripiprazole, while its binding is more potent at 5HT2A, 5HT1A, and adrenergic α1B receptors compared to the same antipsychotic (88). Therefore, it is expected that brexpiprazole would possess less activating properties and would induce fewer akathisia and EPS than aripiprazole (88). Brexpiprazole also has affinity as an antagonist of D3, 5HT2B, 5HT7, and α1A, α1D receptors, moderate affinity for histamine H1 receptors, and low affinity for muscarinic M1 receptors (89).

This antipsychotic is metabolized by CYP2D6 and CYP3A4 isoenzymes and recommended dose adjustments (i.e., 50% reduction) are required for patients with CYP2D6 poor metabolizer status, as approved by both FDA and EMA (3, 90). CYP2D6 poor metabolizers have 47% higher exposure to brexpiprazole vs. extensive metabolizers (90). Also, if patients with this status receive strong or moderate CYP3A4 inhibitors while taking brexpiprazole, the antipsychotic dose should be decreased by 75% (3, 90). The brexpiprazole dose should be reduced by 50% if strong CYP2D6 inhibitors are co-administered, and a quarter of the recommended dose of brexpiprazole is to be administered if strong/moderate CYP2D6 inhibitors and strong/moderate CYP3A4 inhibitors are simultaneously administered to the same patient (3, 90).

If brexpiprazole is co-administered with strong CYP3A4 inducers in a patient already stabilized on this antipsychotic, the daily dose of brexpiprazole will be titrated up to double the recommended dose over 1–2 weeks (3, 90). The daily dose of brexpiprazole may be further increased, but it should not exceed 12 mg/day when used concomitantly with strong CYP3A4 inducers, according to the European product label (90).

Extensive CYP2D6 metabolizers who receive both CYP3A4 and CYP2D6 inhibitors may have a 4-fold to 5-fold increase of the brexpipazole plasma concentration (3, 90); therefore, the reduction to a quarter of the dose is recommended, according to the European product label (90).

The active metabolite, DM-3411, had a comparable inhibitory effect on CYP3A4 and 2D6 with the parent drug, according to *in vitro* studies, which is minimal (91). Brexpiprazole and its active metabolite did not exhibit a significant inhibitory *in vitro* effect on hepatic and renal transporters (organic anion transporting polypeptides- OATP, organic anionic transporter- OAT, organic cationic transporter-OCT, multidrug and toxin extruder- MATE1, and bile salt export pump- BSEP), and brexpiprazole did not affect the function of P-gp and BCRP (91).

Cariprazine is a D3-preferring D3/D2 receptor partial agonist and serotonin 5HT1A receptor partial agonist (92). According to PET studies using [^11^C]-(+)-PHNO as a radioligand, cariprazine has a three times higher affinity for D3 receptors than for D2 receptors, while brexpiprazole did not significantly reduce the availability of D3-receptors *in vivo* (93, 94). Cariprazine has a binding affinity for the D3 receptor three times higher than dopamine itself (K_*i*_ = 0.09 nM vs. 60 nM), and this is an important pharmacodynamic property specific to cariprazine within its class (i.e., brexpiprazole has a K_*i*_ = 1.1 nM, and aripiprazole a K_*i*_ value of 4.6 nM) (95). Therefore, at physiological doses, cariprazine has unique D3 binding properties, unlike other dopamine partial agonists/antagonists whose D3 receptor binding may be reversed by dopamine (93, 94, 96). Cariprazine presents a relatively high affinity for 5HT1A receptors and a low affinity for 5HT2A receptors (92). Also, high affinity for 5HT2B (pure antagonist) and moderate to low affinity for H1 and 5HT2C receptors have been demonstrated for cariprazine during *in vitro* studies (97).

Cariprazine is metabolized in the liver by CYP3A4 and, to a lesser extent, by CYP2D6, to two active metabolites, demethyl- and didesmethyl-cariprazine (DCAR and DDCAR) (98, 99). DCAR is further metabolized to DDCAR by CYP3A4 and 2D6, while DDCAR is hydroxylated by CYP3A4 (99). Both active metabolites have similar properties with the parent drug, although their *in vitro* selectivity for D3 vs. D2 receptors was higher than in the case of cariprazine (99, 100). In rodent models of antipsychotic-like activity, DDCAR was 3- to 10-fold less potent than cariprazine, consistent with the D2 receptor occupancy profile (100). Based on data derived from three phase I trials and ten phase II/III studies in adult patients with schizophrenia or bipolar disorder treated with cariprazine, CYP2D6 metabolizer status was not associated with changes in exposure levels for the active moiety or the two active metabolites (99).

According to the US product label, the dose of cariprazine should be reduced by 50% if co-administered with potent CYP3A4 inhibitors, while the concomitant administration of cariprazine and CYP3A4 inducers is not recommended, according to the SPC of this antipsychotic (101). Based on the European product label, cariprazine’s administration is contraindicated with the concomitant use of strong or moderate CYP3A4 inhibitors or inducers (102). These recommendations are based on the involvement of CYP3A4 isoenzymes in the formation and elimination of the major active metabolites of cariprazine (101, 102).

Detailing the above-mentioned recommendations, the US product label specifies that if strong inhibitors are initiated in patients undergoing treatment with 4.5 mg of cariprazine, its daily dose should be reduced to 1.5 or 3 mg, and if the current dose is 1.5 mg QD, then it should be administered every other day (101). In the case of CYP3A4 inhibitor discontinuation, the cariprazine dose may need to be increased (101). If cariprazine is initiated in a patient already taking a strong CYP3A4 inhibitor, the antipsychotic should be administered on the first and third day at 1.5 mg/day, and from day 4, it should be administered daily 1t 1.5 mg, then increased to a maximum 3 mg/day dose (101). The concomitant initiation of cariprazine and CYP3A4 inhibitors has not been systematically evaluated, and the effect on the active drug and major metabolites is unclear (101). As already mentioned, the European product label clearly specifies that concomitant administration of strong/moderate CYP3A4 inhibitors/inducers is contraindicated when cariprazine is used (102).

Regarding other targets potentially influenced by genetic variability, neither cariprazine nor its metabolites are substrates of P-gp, organic anion transporting polypeptides 1B1 and 1B3 (OATP1B1 and OATP1B3), or the breast cancer resistance protein (BCRP) (101).

Lumateperone is a novel agent with D2 presynaptic partial agonism and postsynaptic antagonism, but it also has 5HT2A receptors antagonism properties, inhibits the serotonin reuptake transporter, and has the capacity to modulate the GluN2B subunit of the NMDA receptor (63). Lumateperone has more than 60 times higher potency for 5HT2A receptors than for D2 receptors, which explains the lower risk for EPS (63). The low affinity of this antipsychotic for α1 and H1 receptors correlates with a good tolerability profile (63).

It is extensively metabolized, and more than 20 metabolites have been identified, with CYP3A4, 2C8, 1C1, 1B10, 1C4, and 1A2 participating in this process (63, 103). Also, lumateperone is a substrate for uridine 5′-diphospho-glucuronosyltransferases (UGT), which became important if inhibitors of these enzymes are coadministered (e.g., valproate); therefore, the concomitant administration of lumateperone and valproate is not recommended (63). P-gp and BCRP are not involved in the metabolization of lumateperone (103). If CYP3A4 inducers are already administered in a patient, lumateperone’s initiation should be avoided (103). Also, if moderate or strong CYP3A4 inhibitors are administered, concomitant use of lumateperone should be avoided (103). Concomitant use of lumateperone and UGT inhibitors may increase exposure to the antipsychotic and/or its metabolites (103).

Pimavanserin is an atypical antipsychotic which acts as an inverse agonist/antagonist of 5HT2A and 5HT2C receptors, without significant interference with D2 receptors (64). Its exact mechanism of action is unknown, but its affinity for other receptors (including histamine, muscarinic, or adrenergic), except for serotonergic 2A and 2C, is very low (64).

If it is co-administered with strong CYP3A4 inhibitors, its dose should be reduced by 50% (104). Strong CYP3A4 inducers co-administered with pimavanserin indicate the need for monitoring reduced efficacy, which may require an increased pimavanserin daily dose (104).

Pimavanserin is predominantly metabolized by CYP3A4 and 3A5 and, to a lesser extent, by CYP2J2, 2D6, and other CYP and FMO enzymes (104). The major metabolite (AC-279) results from the intervention of CYP3A4 (104). Transporters do not intervene significantly affect the disposition of pimavanserin (104).

Regarding the recommendations of the pharmacogenetic guidelines, the CPIC includes references to certain antidepressants (e.g., atomoxetine) and antiepileptics (e.g., carbamazepine/oxcarbazepine), but the recommendations about antipsychotics (including aripiprazole and brexpiprazole) appear to be “in progress,” according to the official site of this organization (105).

The DPWG guidelines include recommendations for aripiprazole and brexpiprazole (106). Based on these guidelines, the dose of aripiprazole should be reduced in poor metabolizers of CYP2D6, and no more than 10 mg/day or 300 mg/month can be administered, representing 68–75% of the standard maximum dose of aripiprazole (106). For brexpiprazole, in poor metabolizers, these guidelines recommend half of the standard dose (106).

The International Society of Psychiatric Genetics (ISPG) Consensus states that the use of pharmacogenomic testing is supported by evidence in the literature, prescribing guidelines, and product labels, in the case of two cytochrome-P450 genes, i.e., 2D6 and 2C19 (107, 108). Also, evaluation of the human leukocyte antigen (HLA) genes in patients undergoing treatment with carbamazepine (HLA-A and B), oxcarbazepine (HLA-B), and phenytoin (CYP2C9, HLA-B) is granted based on currently available evidence (108). In the case of valproate, pharmacogenetic testing is helpful for certain genes, i.e., POLG, OTC, and CSP1, when a mitochondrial disorder or a urea cycle disorder is suspected (108).

## 6. Conclusion

The pharmacogenetic-based approach in patients with SPDs has as its main rationale the improvement of therapeutic management, by eliminating the traditional empirical strategy in the selection of pharmacological agents. The importance of exploring the pharmacogenetic characteristics of the new-generation antipsychotics derives from multiple clinical perspectives, e.g., the possible prediction of treatment efficacy or lack of responsivity, the anticipation of specific adverse events, the need to increase treatment adherence and patients’ quality of life, etc. In order to attain these objectives, the current scoping review investigated available data collected from primary and secondary sources, and from the SPCs, allowing for extracting conclusions based on both clinical and preclinical data, *in vivo* and *in vitro* studies.

Based on the reviewed sources, *aripiprazole* benefit from the most relevant data about the impact of gene variability on its pharmacokinetics and pharmacodynamics, with significant consequences on this antipsychotic’s efficacy and tolerability (68, 69, 71, 80, 82, 85, 87). The determination of the CYP2D6 metabolizer status is important when administering aripiprazole, both as monotherapy and associated with other drugs. FDA, EMA, and DPWG guidelines formulated recommendations regarding the dosage of aripiprazole oral and LAI, and data from case reports, clinical trials, and reviews support this perspective. The co-administration of CYP3A4 strong inhibitors needs supplementary monitoring and dose adjustment of the aripiprazole regimen. Other significant pharmacogenetic observations refer to the CYP3A5 and P-gp genotype on the risk of adverse events, but they are based on fewer good-quality data (71). Allelic variability in genes encoding D2, D3, 5HT2A, 5HT2C receptors, COMT, BDNF, and dopamine transporter DAT1 was also associated with different adverse events (e.g., dizziness, EPS, akathisia, etc.) or variation in the clinical efficacy (71, 80, 83, 85, 87).

*Brexpiprazole* also benefits from specific recommendations regarding the CYP2D6 metabolizer status and the association of this antipsychotic with strong/moderate CYP2D6 or CYP3A4 inhibitors. These recommendations were formulated by FDA and DPWG guidelines (3, 90, 106). Unfortunately, no data were found about the impact of genetic variations on the pharmacodynamics of this antipsychotic, and about the possible correlations between the genotype and the tolerability profile.

*Cariprazine* benefits from FDA and EMA recommendations regarding possible pharmacokinetic interactions with strong CYP3A4 inhibitors or inducers (101, 102), but pharmacogenomics data is sparse. Based on the available evidence, it seems that CYP2D6 metabolizer status has no significant impact on the exposure levels of cariprazine or its active metabolites (101, 102).

Data on *lumateperone* and *pimavanserin* are even more scarce and, although pharmacokinetic recommendations are available about potential interaction with CYP3A4 inducers or inhibitors (103, 104), concrete pharmacogenetic studies with these two antipsychotics could not be found.

There are a few limitations of the current review, starting with the fact that it was not based on a systematic strategy; therefore, possible sources could have been missed. Due to its narrative nature, the review did not include a scoring procedure for the quality of evidence, and all the reports were considered equally relevant to the objective of the paper. It must also be noted that the review evaluated studies enrolling healthy volunteers and not only patients with SPD, but this was allowed due to the need to have control groups in the context of genetic variability across diverse populations.

Besides the methodological limitations of this review, a set of limitations regarding to the pharmacogenetic research should be mentioned. First, the association of single genetic polymorphisms with specific clinical features (in efficacy and/or tolerability studies) may explain the variability of results across different sources, because these studies underestimate the complexity of the relationship between genetic variants (109). The pharmacogenomics studies exploring antipsychotics are limited in their conclusions due to the lack of information about the functional impact of genetic variants, except for the already well-defined pharmacogenes (110). It must also be observed that there are multiple inter-individual genetic differences, besides those related to the ethnic and race variables, including in the domain of pharmacogenes, which significantly raise the heterogeneity of study groups (109, 110). The phenomenon of phenoconversion, already mentioned in the second section of the review (29), is another factor that limits the clinical utility of genotyping, for example in the case of CYP2D6 isoenzyme. But this phenomenon is the main reason why data about the pharmacokinetics of each antipsychotic was mentioned, and why the case manager should be aware of all the potential pharmacokinetics interactions when co-administering drugs in these patients. For clinical practice, it is important for psychiatrists to be aware of the possible CYP2D6 phenotypic translations.

In conclusion, it is hoped that future studies will validate the importance of pharmacogenomic testing for choosing an optimal pharmacological treatment for individual patients, paving the path for personalized medicine. These studies will have to explore the clinical and pharmacoeconomic impact of pharmacogenomic-guided treatments in SPD, especially SSD, MDD, and bipolar disorders. Also, the validation through prospective studies of diagnostic and prognostic algorithms combining genomic information with transcriptomics, proteomics, metabolomics data, neuroimaging, and clinical characteristics in patients with SPD would significantly increase the probability of implementing personalized psychiatric treatments. Extensive *pharmacogenetics* studies are needed to determine the impact of specific gene effects and *pharmacogenomics* trials (based on genome-wide association research) to explore the complex interactions between genes in determining the responsivity/tolerability to the pharmacological agents. It is expected that these data will be included in future treatment guidelines and diagnostic algorithms for patients with SPD, improving their functional prognosis.

## Author contributions

The author confirms being the sole contributor of this work and has approved it for publication.
